# Supplementary material for: How liquids charge the superhydrophobic surfaces
Source: Nat Commun. 2024 Jun 4;15:4762. doi: 10.1038/s41467-024-49088-1 (PMC11150272; doi:10.1038/s41467-024-49088-1)
Supplement: Supplementary file 1 — Supplementary Information [file 41467_2024_49088_MOESM1_ESM.pdf]

Supplementary Information

How liquids charge the superhydrophobic surfaces

Yuankai Jin[1,2], Siyan Yang[1], Mingzi Sun[3], Shouwei Gao[1],Yaqi Cheng[2], Chenyang Wu[2], Zhenyu Xu[2], Yunting Guo[2], Wanghuai Xu[4], Xuefeng Gao[5], Steven Wang[2], Bolong Huang[3,*], and Zuankai Wang[1,6,*]

[1]Department of Mechanical Engineering, The Hong Kong Polytechnic University, Hong Kong SAR, P.R. China.

[2]Department of Mechanical Engineering, City University of Hong Kong, Hong Kong SAR, P.R. China.

[3]Department of Applied Biology and Chemical Technology, The Hong Kong Polytechnic University, Hong Kong SAR, P.R. China.

[4]Department of Electrical and Electronic Engineering, The Hong Kong Polytechnic University, Hong Kong SAR, P.R. China.

[5]Suzhou Institute of Nano-Tech and Nano-Bionics, Chinese Academy of Sciences, Suzhou, P.R. China.

[6]Research Centre for Nature-Inspired Science and Engineering, The Hong Kong Polytechnic University, Hong Kong SAR, P.R. China.

* Bolong Huang (bhuang@polyu.edu.hk), Zuankai Wang (zk.wang@polyu.edu.hk)

Supplementary Note 1. Calculation of the static charges in coalescence-induced jumping droplets.

The static charges in the coalescence-induced jumping droplet are calculated based on the force balance under the electric field. Supplementary Figure 1b shows that the randomly selected jumping droplet experiences a uniform ascending motion with a velocity (v) of ~ 11.9 mm s^{-1}, and the droplet radius (R) is ~ 10.3 um, determined from the videos captured by a high-speed camera. Such uniform motion of the droplet illustrates its force balance, that is $q\mathbf{E} + \mathbf{F}_B = \mathbf{F}_D + mg$ (Supplementary Figure 2), where $q\mathbf{E}$, $\mathbf{F}_B$, $\mathbf{F}_D$, and mg is the electrostatic force, buoyancy force, drag force, and gravity, respectively. Among that, the buoyancy force is negligible due to the extremely low density of vapor phases (ρ_v = 0.0269 kg m^{-3}). The drag force can be calculated according to the Stokes' Law, $\mathbf{F}_D = 6{\cdot}\pi{\cdot}\mu{\cdot}v{\cdot}R$, where μ is the vapor viscosity (18.09×10^{-6} Pa s, a value in ambient environments). Note that it is deemed appropriate to use a Stokes drag due to the low Reynolds numbers of the droplet motion. The gravity can be calculated by using the equation, $\mathrm{mg} = \frac{4}{3}\pi\rho_{\omega}R^3 g$, where ρ_{ω} is the density of liquid water (998 kg m^{-3}). Under a downward electric field (with a strength of ~ 15 kV m^{-1}), the charges in one randomly selected droplet (R of ~ 10.3 μm and a terminal v of ~ 11.9 mm s^{-1}) are calculated as -5.57×10^{-15} C, *i.e.*, -5.57 fC.

Supplementary Note 2. Calculation Setup.

To study the charge generation during liquid-solid CE occurring on superhydrophobic surfaces, we have applied the density functional theory calculations within CASTEP packages[1]. To describe the exchange-correlation interactions accurately, we have used the hybrid functional for all the calculations to alleviate the self-interaction error. For functionals, we have applied the HSE06 functionals with norm-conserving pseudopotentials and the cutoff energy has been set to 630 eV. In addition, the Broyden-Fletcher-Goldfarb-Shannon (BFGS) algorithm[2] with EDFT as the electronic minimization. For the k-points, we have set the fine quality for all the energy minimizations. We have constructed DT and FDT in vertical alignments. For all the geometry optimizations in this work, the following convergence criteria have to be satisfied, including the Hellmann-Feynman forces should not exceed 0.001 eV $Å^{-1}$, the total energy difference, and the inter-ionic displacement should be less than 5×10^{-5} eV per atom and 0.005 Å, respectively.

Supplementary Note 3. Measurement of static charges in coalescence-induced jumping droplets.

We used the setup illustrated in Supplementary Figure 4a to measure the static charges in droplets jumping from the superhydrophobic surfaces with various k values. The setup design enables the condensation of vapor from the environment due to the surfaces that are adhered to a cooler plate. The condensate droplets could coalesce on cold superhydrophobic surfaces and then jump to fall into the Faraday cup. The Faraday cup, which is connected to a nanocoulomb meter, can record droplet charges and further output the results to an oscilloscope (RTE 1054, Rohde & Schwarz). Supplementary Figure 4b shows the recorded charges in condensate droplets within a collection time of 120 seconds. We find that the k values of surfaces could tailor the polarity and magnitude of generated charges during CE. The maximum negative and positive electrostatic charges are observed at $k = 0$ (superhydrophobic DT surfaces) and $k = 1$ (superhydrophobic FDT surfaces), respectively. The polarity of the charges switches between negative and positive when k values range from 0.1 to 0.15.

Supplementary Note 4. The influence of droplet impact dynamic on the CE between liquids and superhydrophobic surfaces.

Droplet impact dynamic on superhydrophobic surfaces are determined by the tilt angle and dropping heights (Supplementary Figure 7a) because these two parameters affect the maximum spreading area (A_{max} in Supplementary Figure 7b) and sliding length (Supplementary Figure 7c), which further affect the generated charges. Supplementary Figure 7d shows that A_{max} (left y-axis) decreases while the sliding length (right a-axis) increases with the increase of tilt angles of the superhydrophobic surfaces. We find that maximum charge generation in water at the tilt angles of 45° (Supplementary Figure 7e), with neither the largest A_{max} nor largest sliding length, indicating the coeffect of A_{max} and sliding length on charge generation. Regarding the effect of dropping height on charge generation, Supplementary Figure 7f shows that the magnitude of generated charges gradually increases and then saturates with the increase of dropping height. In a large dropping height (for example, 4.5 cm), the droplet bouncing off the superhydrophobic surface typically fragments into two daughter droplets, during which new types of charges (may not be triboelectric charges) may be generated and thus, affect the quantification of triboelectric charges.

Supplementary Note 5. Stable charging ability of superhydrophobic surfaces in liquid-solid CE.

The main text has already presented that superhydrophobic surfaces maintain a stable charging ability even after exposure to ion-enriched liquids, including acid, alkali, and salt aqueous solutions, which is attributed to the absence of ion residue on superhydrophobic surfaces. Here, we further consolidate this point by revealing time-involved linearly accumulated liquid charges.

For previously reported liquid-solid CE, the hydrophilic/hydrophobic surfaces usually undergo a charging ability degradation during contact with the liquids due to the ion transfer.[3, 4] The ions from the liquids could continuously adsorb on the solid surfaces during CE, until saturation (illustrated in Supplementary Figure 8a). These ions also change the surface potential (or work function) of solid surfaces, which eliminates the original work function difference between the pristine solid surface and liquid medium, thereby suppressing the electron transfer during CE. In other words, with the CE proceeding, both electron and ion transfer are suppressed by the gradually adsorbed ions on the surfaces, indicating a deterioration of charging ability. The surfaces with adsorbed ions can also be regarded as the pre-charged surfaces, which have been demonstrated to influence the charge generation during CE.[5] However, in the case of CE between liquids and superhydrophobic surfaces, generated charges exhibit a linear increase over time (as shown in Supplementary Figure 8b), indicating a stable charging ability of superhydrophobic surfaces. This stable charging ability is also evident in the CE between coalescence-induced jumping droplets and the superhydrophobic surfaces, as depicted in Supplementary Figure 4b.

Supplementary Note 6. pH independence of CE between liquids and superhydrophobic surfaces.

Liquid-solid CE occurring on superhydrophobic surfaces shows independence from the liquid pH. Previous studies have proven that charge generation during liquid-solid CE is usually dependent on liquid pH, which manifests that the charge polarity is determined by the pH of the liquids and the isoelectric point of the solid surfaces. Here, the isoelectric point refers to a critical pH value (generally around 3-4) for solid surfaces at which the solid surfaces become electrically neutral.[6, 7] When the pH of the liquids (*e.g.,* water or alkali solution) is higher than the isoelectric point of solid surfaces, the

generated charges on the solid surfaces are always negative.[8] Conversely, when the pH of the liquids (acidic solution) is lower than the isoelectric point, the charges on the solid surfaces are always positive.[9, 10] These results arise from the competition between cations and anions for adsorption during CE. When the pH of the liquids is lower or higher than the isoelectric point, the predominant ions on the solid surfaces are cations or anions, respectively,[6, 7] thereby determining the charge polarity during liquid-solid CE. However, this conventional rule is not applicable to the CE occurring on superhydrophobic surfaces since ion transfer is not involved in such a CE process. Though the isoelectric point of superhydrophobic FDT and DT surfaces is 4.62 and 4.22 (Supplementary Figure 9), respectively, the liquids, regardless of their pH values, including water (typically at pH 7), acid solution (pH=2) and alkali solution (pH=12), consistently acquire positive charges from FDT surfaces and negative charges from DT surfaces (Supplementary Figure 10). Like that in the CE between water and superhydrophobic surfaces, there is also a linear correlation between surface work functions and charges in both acidic and alkaline aqueous solutions. These results collectively indicate that liquid-solid CE occurring on superhydrophobic surfaces is independent of the liquid pH.

Figures

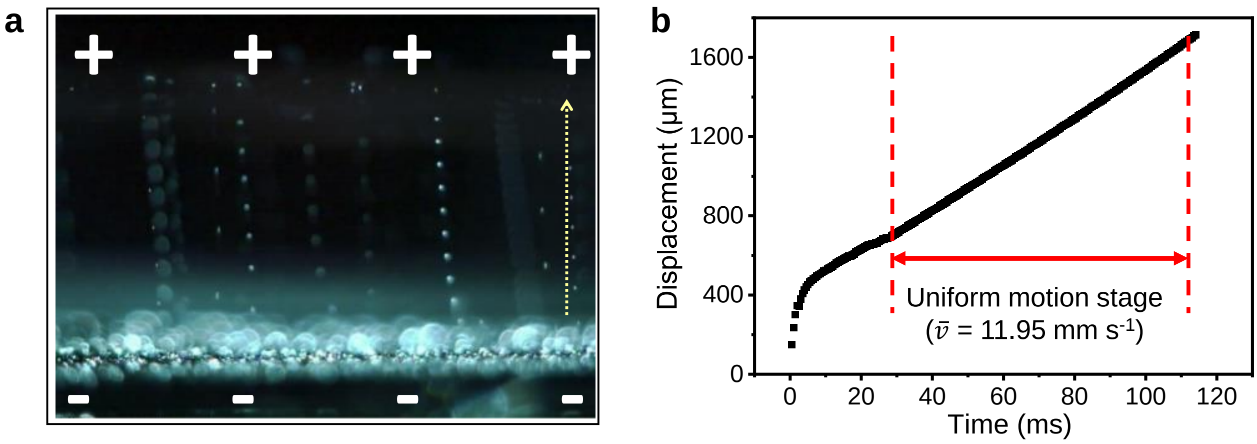

Supplementary Figure 1. The motion of the condensate droplets jumping from the superhydrophobic DT surfaces under an electric field directed downward. a Time-lapse image showing the jumping droplets continuously ascending towards the top surface, indicating the generation of negative charges in the droplets. **b** The displacement of the jumping droplet as a function of its traveling time.

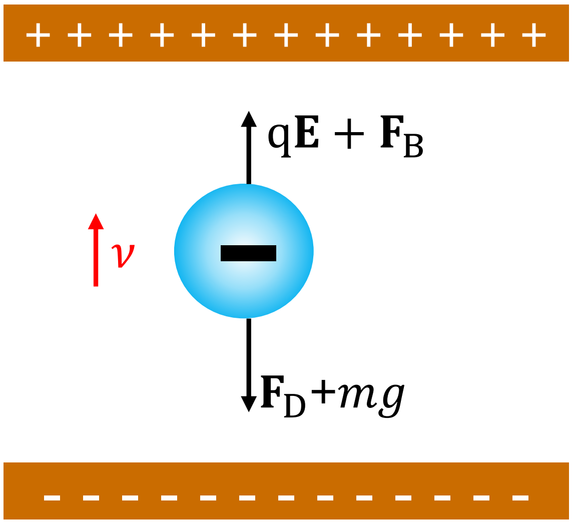

Supplementary Figure 2. Force analysis for charged liquid droplets that ascend under the influence of an downward-directed electric field. Here, $q\mathbf{E}$, $\mathbf{F}_B$, $\mathbf{F}_D$, mg, is the electrostatic force, buoyancy force, drag force, and gravity, respectively. v denotes the upward movement of the droplets.

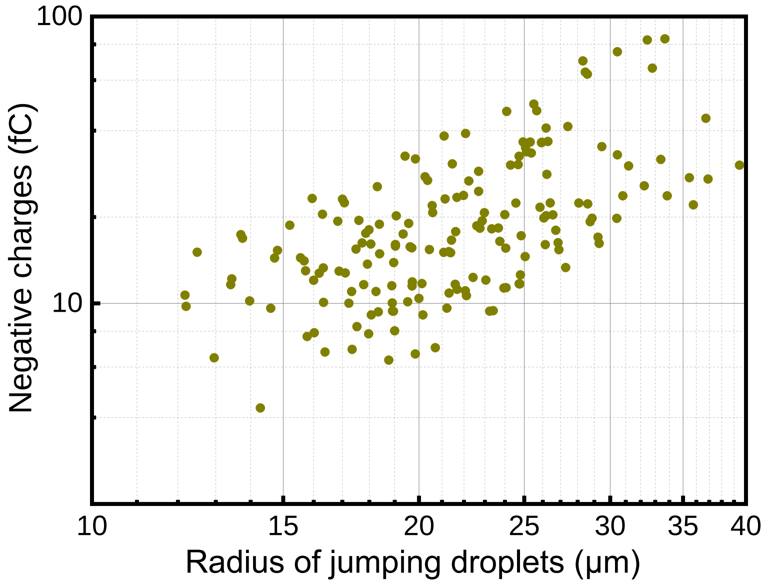

Supplementary Figure 3. Radius distribution of droplets jumping from superhydrophobic DT surfaces and corresponding magnitude of negative charges in droplets.

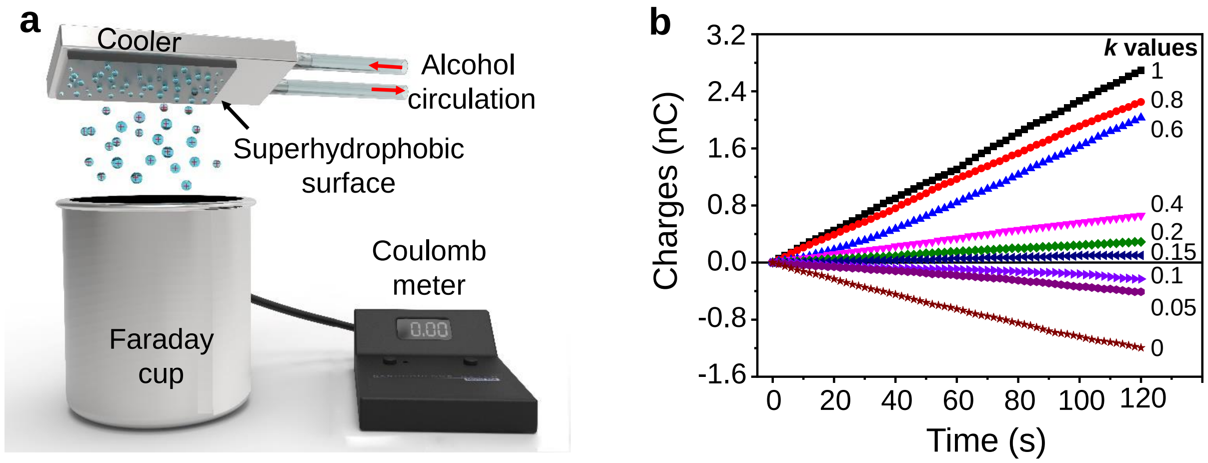

Supplementary Figure 4. Measurement of static charges in coalescence-induced jumping droplets. a The schematic diagram for the setup that enables the recording of the static charges generated in CE between superhydrophobic surfaces and coalescence-induced jumping droplets. The superhydrophobic samples adhered to the cooler are cooled down to 0.5 °C by circulating the alcohol. The condensate droplets are generated on the cold surfaces and then undergo coalescence-induced jumping. The jumping droplets are collected in a Faraday cup, allowing for the recording of the charges by a Coulomb meter. **b** The accumulation of static charges in jumping droplets from the superhydrophobic surfaces with varied *k* values, for 120 seconds.

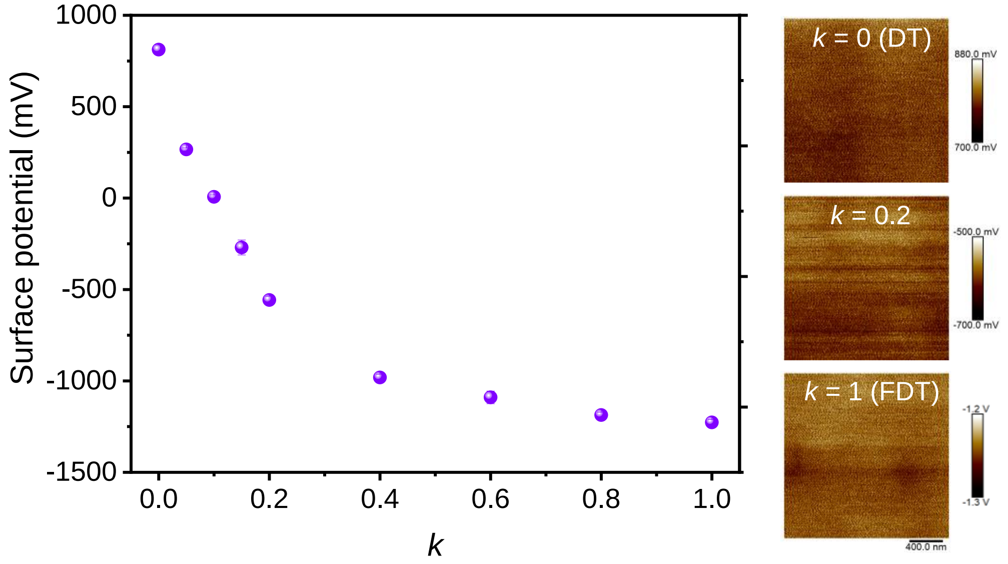

Supplementary Figure 5. The influence of *k* values on surface potential of superhydrophobic surfaces and KPFM images of the surface with selected *k* values. The error bars are based on the SD values of three tests, and the error bars are too small to allow clear differentiation.

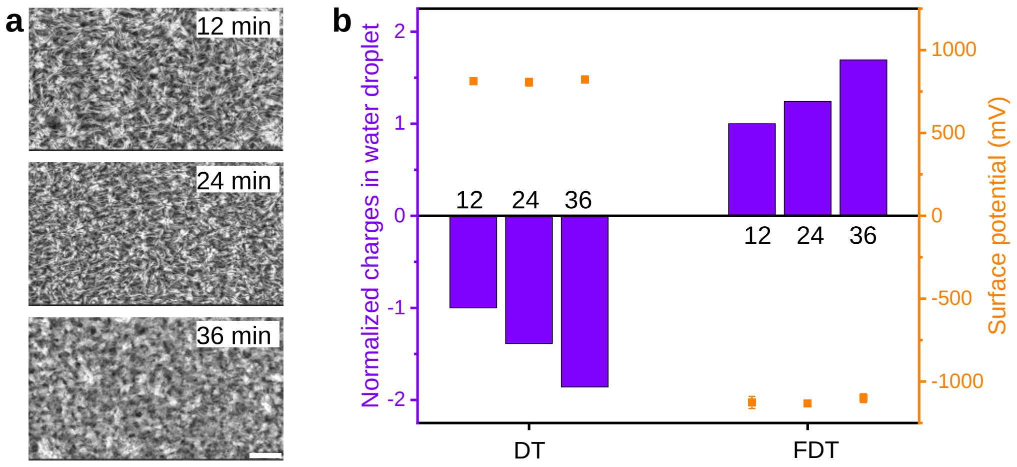

Supplementary Figure 6. The influence of surface morphology on liquid-solid CE. a Morphology of superhydrophobic surfaces with different etching time. With the etching time increase from 12 to 36 minutes, the surface structure undergoes a gradual transformation from nano-grass to nano-protrusion, leading to the larger liquid-solid contact area and more static charges generation in droplets during CE (left y-axis in **b**). However, the surface potentials remain consistent across the surfaces with different etching time (right y-axis in **b**). The scale bar is 5 μm.

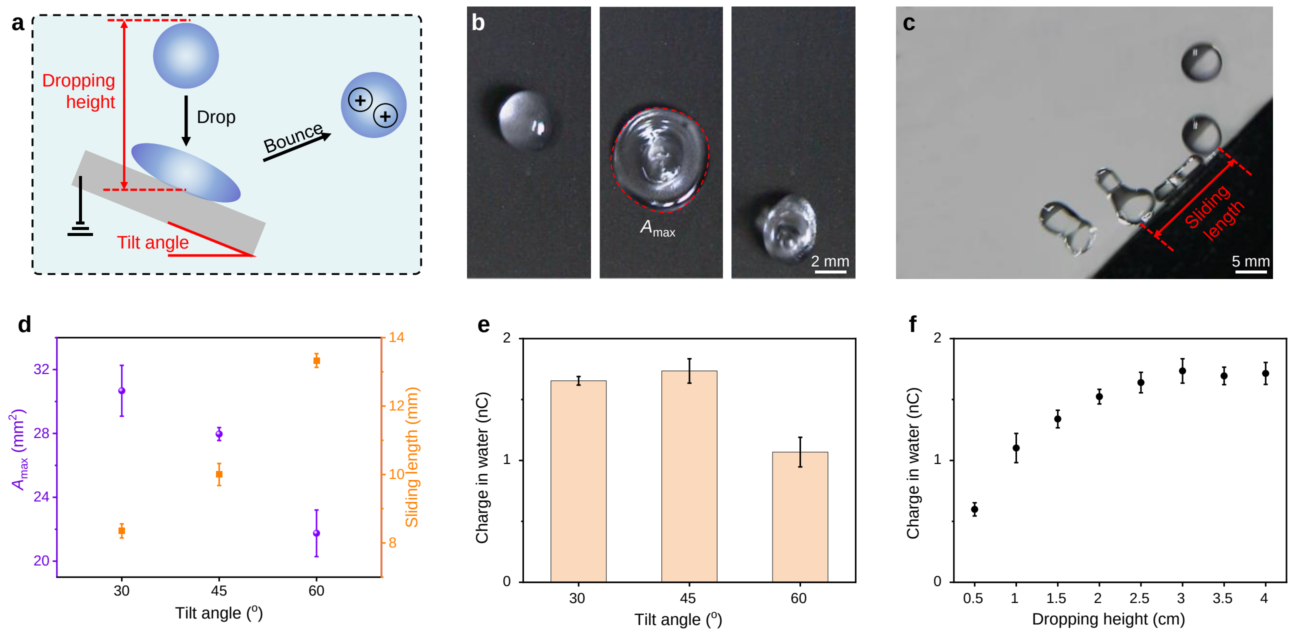

Supplementary Figure 7. Influence of droplet impact dynamic on charge generation during CE between water and superhydrophobic surfaces. **a** Schematic diagram illustrating the parameters that affect droplet impact dynamic. **b** The optical images of the water droplet impact on the tilted superhydrophobic surfaces. A_{max} denotes the maximum spreading area of the water droplet. **c** A time-lapsed droplet trajectory that records the sliding process of droplets after impact on the surfaces. **d** The A_{max} and sliding length of impacted droplets on the superhydrophobic surfaces with varied tilt angles. The data are derived from figures and videos that were analyzed using the ImageJ software. **e** Droplet charges generated during CE between water and superhydrophobic FDT surfaces with varied tilt angles. The dropping height in **b-e** is 3 cm. **f** The variation of charges generated in water as the function of dropping height. The magnitude of generated charges gradually saturates with the increase of dropping height. The error bars in **d-f** are based on the SD values of three tests.

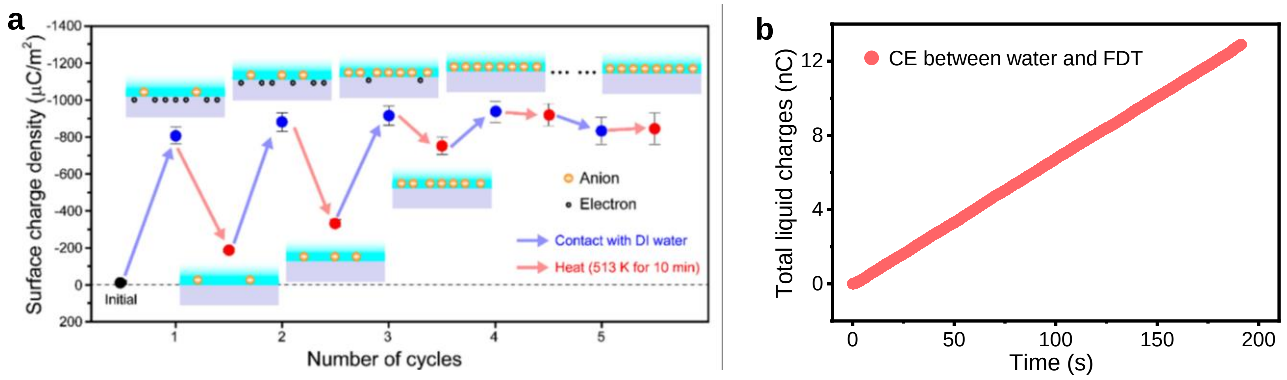

Supplementary Figure 8. Comparison of charging ability on surfaces with different wettability. **a** Charge density on the (hydrophilic) SiO_2 surfaces in the charging-heating cycle tests. With the cycles increasing, the charges (*i.e.*, anions) on SiO_2 surfaces gradually saturate, which simultaneously suppresses the electron transfer between SiO_2 and water. Reprinted with permission from ref.[11]. Copyright 2022 American Chemical Society. **b** The time-evolved accumulation of charges in water droplets during CE occurring on superhydrophobic FDT surfaces. The linear growth of charges implies that each water droplet maintains relatively constant charges, demonstrating that the electrifying ability of FDT surfaces is unaffected by the impacts of liquid droplets. Here, the droplet dripping frequency is ~ 6.7 Hz. The droplet charges are measured using a Faraday cup and nanocoulomb meter, then output to the oscilloscope.

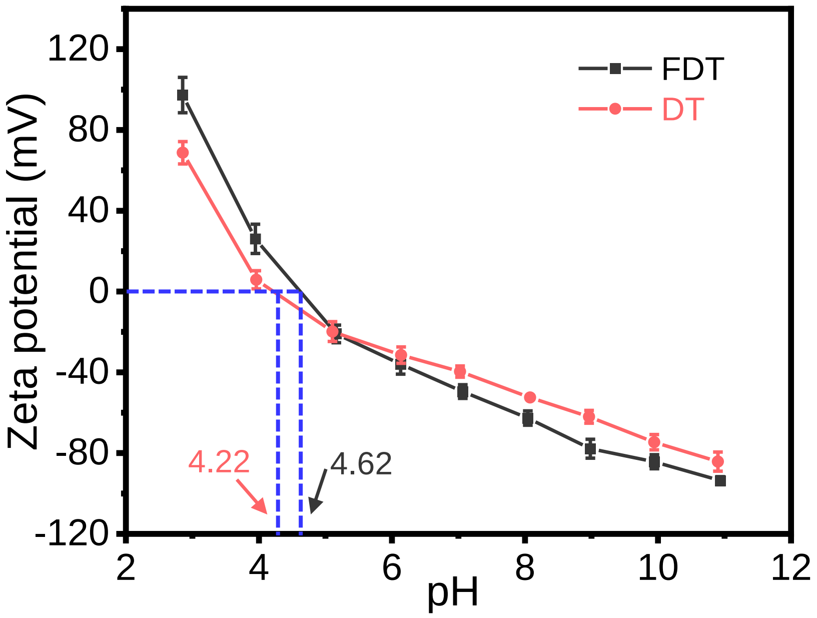

Supplementary Figure 9. Zeta potential curve of superhydrophobic FDT and DT surfaces in 1 mM of KCl solution. The curves are measured by a commercial electrokinetic analyzer (SurPASS, Anton-Paar GmbH). The figure denotes that the isoelectric point of superhydrophobic FDT and DT surfaces is 4.62 and 4.22, respectively. The error bars are based on the SD values of four tests.

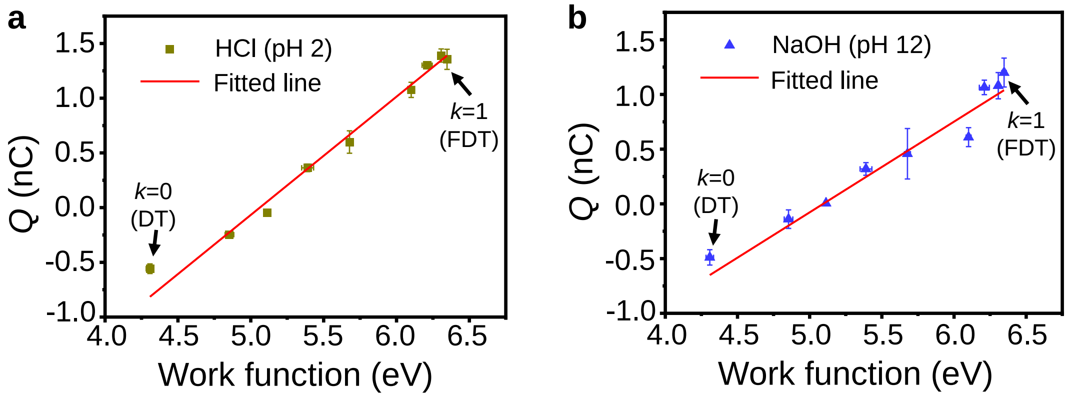

Supplementary Figure 10. The correlation between work functions of superhydrophobic surfaces·with varied *k* and the charges in acid solutions (a) and alkali solutions (b). The error bars are based on the SD values of three tests.

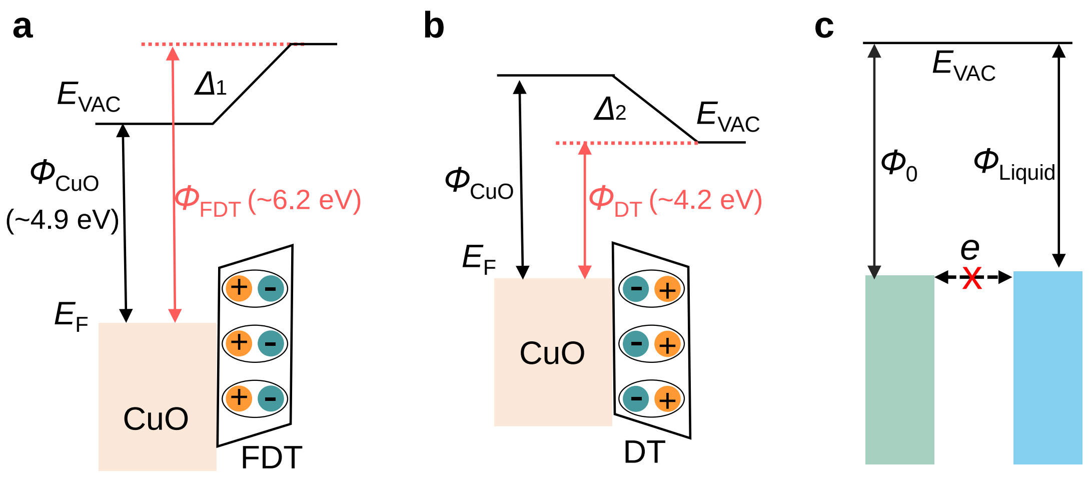

Supplementary Figure 11. Sketch for the capability of FDT and DT to regulate work functions and electron transfer. a FDT with trifluoromethyl group increases the surface work function $\emptyset_{FDT} = \emptyset_{CuO} + \Delta_1$ by shifting the vacuum energy level, E_{VAC}. **b** DT with methyl group decreases the surface work function $\emptyset_{DT} = \emptyset_{CuO} - \Delta_2$. **c** The electron transfer is blocked when the solid surfaces and liquids have identical work functions, leading to no charge generation between liquid-solid CE.

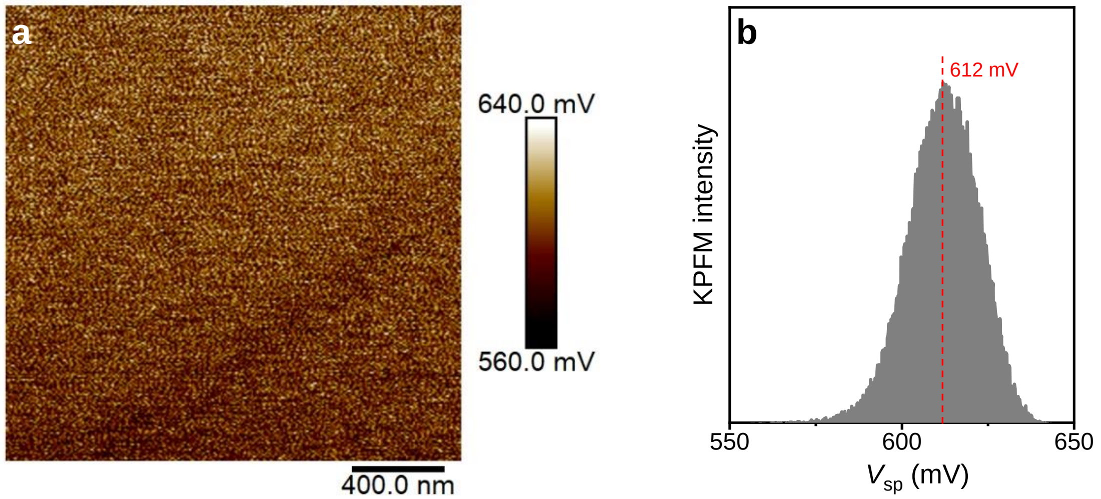

Supplementary Figure 12. KPFM image of HOPG (a) and corresponding histogram of surface potential (b). The value corresponding to the most significant intensity (*i.e.*, the peak of the histogram, 612 mV) is the value of surface potential.

References

1. Clark, S. J.; Segall, M. D.; Pickard, C. J.; Hasnip, P. J.; Probert, M. J.; Refson, K.; Payne, M. C. First principles methods using CASTEP. *Zeitschrift Fur Kristallographie* ***220***, 567-570 (2005).
2. Head, John D.; Zerner, Michael C. A Broyden—Fletcher—Goldfarb—Shanno optimization procedure for molecular geometries. *Chem. Phys. Lett.* ***122***, 264-270 (1985).
3. Lin, S.; Xu, L.; Chi Wang, A.; Wang, Z. L. Quantifying electron-transfer in liquid-solid contact electrification and the formation of electric double-layer. *Nat. Commun.* ***11***, 399 (2020).
4. Zhan, F.; Wang, A. C.; Xu, L.; Lin, S.; Shao, J.; Chen, X.; Wang, Z. L. Electron Transfer as a Liquid Droplet Contacting a Polymer Surface. *ACS Nano* ***14***, 17565-17573 (2020).
5. Tang, Zhen; Lin, Shiquan; Wang, Zhong Lin Effect of Surface Pre-Charging and Electric Field on the Contact Electrification between Liquid and Solid. *J. Phys. Chem. C* ***126***, 8897-8905 (2022).
6. Beattie, J. K. The intrinsic charge on hydrophobic microfluidic substrates. *Lab Chip* ***6***, 1409-1411 (2006).
7. Beattie, James K., The Intrinsic Charge at the Hydrophobe/Water Interface. In *Colloid Stability*, 2010; pp 153-164.
8. Burgo, Thiago A. L.; Galembeck, Fernando; Pollack, Gerald H. Where is water in the triboelectric series? *J. Electrostat.* ***80***, 30-33 (2016).
9. Zimmermann, R.; Dukhin, S.; Werner, C. Electrokinetic measurements reveal interfacial charge at polymer films caused by simple electrolyte ions. *J. Phys. Chem. B* ***105***, 8544-8549 (2001).
10. Nie, J.; Ren, Z.; Xu, L.; Lin, S.; Zhan, F.; Chen, X.; Wang, Z. L. Probing Contact-Electrification-Induced Electron and Ion Transfers at a Liquid-Solid Interface. *Adv. Mater.* ***32***, e1905696 (2020).
11. Lin, S.; Chen, X.; Wang, Z. L. Contact Electrification at the Liquid-Solid Interface. *Chem. Rev.* ***122***, 5209-5232 (2022).
